# Study on the Characteristics of Small-Molecule Kinase Inhibitors-Related Drug-Induced Liver Injury

**DOI:** 10.3389/fphar.2022.838397

**Published:** 2022-04-21

**Authors:** Huiqun Dong, Jia You, Yu Zhao, Danhua Zheng, Yi Zhong, Gaozheng Li, Zuquan Weng, Heng Luo, Shan Jiang

**Affiliations:** ^1^ College of Biological Science and Engineering, Fuzhou University, Fuzhou, China; ^2^ Department of Hepatology, Hepatology Research Institute, The First Affiliated Hospital, Fujian Medical University, Fuzhou, China; ^3^ College of Mathematics and Computer Science, Fuzhou University, Fuzhou, China; ^4^ MetaNovas Biotech Inc., Foster City, CA, United States; ^5^ Department of Vascular Thyroid Surgery, Affiliated Union Hospital, Fujian Medical University, Fuzhou, China

**Keywords:** KIS, DILI, defined daily dose, molecular weight, octanol–water partition coefficient, liver metabolism, docking, drug properties

## Abstract

**Background and Aim:** More than half of the small-molecule kinase inhibitors (KIs) induced liver injury clinically. Meanwhile, studies have shown a close relationship between mitochondrial damage and drug-induced liver injury (DILI). We aimed to study KIs and the binding between drugs and mitochondrial proteins to find factors related to DILI occurrence.

**Methods:** A total of 1,223 oral FDA-approved drugs were collected and analyzed, including 44 KIs. Fisher’s exact test was used to analyze DILI potential and risk of different factors. A total of 187 human mitochondrial proteins were further collected, and high-throughput molecular docking was performed between human mitochondrial proteins and drugs in the data set. The molecular dynamics simulation was used to optimize and evaluate the dynamic binding behavior of the selected mitochondrial protein/KI complexes.

**Results:** The possibility of KIs to produce DILI is much higher than that of other types (OR = 46.89, *p* = 9.28E-13). A few DILI risk factors were identified, including molecular weight (MW) between 400 and 600, the defined daily dose (DDD) ≥ 100 mg/day, the octanol–water partition coefficient (LogP) ≥ 3, and the degree of liver metabolism (LM) more than 50%. Drugs that met this combination of rules were found to have a higher DILI risk than controls (OR = 8.28, *p* = 4.82E-05) and were more likely to cause severe DILI (OR = 8.26, *p* = 5.06E-04). The docking results showed that KIs had a significant higher affinity with human mitochondrial proteins (*p* = 4.19E-11) than other drug types. Furthermore, the five proteins with the lowest docking score were selected for molecular dynamics simulation, and the smallest fluctuation of the backbone RMSD curve was found in the protein 5FS8/KI complexes, which indicated the best stability of the protein 5FS8 bound to KIs.

**Conclusions:** KIs were found to have the highest odds ratio of causing DILI. MW was significantly related to the production of DILI, and the average docking scores of KI drugs were found to be significantly different from other classes. Further analysis identified the top binding mitochondrial proteins for KIs, and specific binding sites were analyzed. The optimization of molecular docking results by molecular dynamics simulation may contribute to further studying the mechanism of DILI.

## Introduction

Drug-induced liver injury (DILI) refers to the unexpected harm to the liver caused by commonly used drugs (Andrade et al., 2019). DILI is the major cause of acute liver failure, which impacts patient health and makes it challenging for drug development (Björnsson, 2010), as severe DILI may lead to drug withdrawal from the market (Chan and Benet, 2017). Analyzing the risk factors of DILI can help reduce the occurrence of this side effect and benefit patient health.

It has been reported that the DILI occurrence was associated with the drug types (Sunil Kumar et al., 2021). Anti-tumor drugs, heparin, antibacterial drugs, and anti-tuberculosis drugs are the most common drug categories that cause DILI in clinical practice (Meier et al., 2005). In recent years, Chinese herbal medicines and antibiotics have also been reported with DILI association (Song et al., 2020). Among the drugs approved by the Food and Drug Administration (FDA), the proportion of anti-cancer drugs rose sharply from 2009 to 2017 (Batta et al., 2020), and small-molecule kinase inhibitors (KIs) have become more popular (Bayazeid and Rahman, 2021). From 2016 to 2018, 53% of new anti-cancer drugs approved by the FDA were oral protein KIs (Ribeiro et al., 2020). A total of 53 small-molecule KIs have been approved by October 2019 (Shi et al., 2020). KIs mainly target the tyrosine protein kinases to achieve therapeutic functions (Sun et al., 2017), and more recent reports discovered additional targets including serine/threonine protein kinases and even lipokinases (Levitzki, 2013; Wu et al., 2016). Despite their anti-cancer effectiveness, KIs may cause DILI as a major safety problem (Shi et al., 2020). According to existing reports, more than half of the FDA-approved 53 KIs caused DILI in clinical observation (Jiang et al., 2021). Analyzing the characteristics of KIs may help to reveal the risk factors associated with DILI.

The occurrence of DILI is related to factors associated with the etiology, the host, and the environment (Garcia-Cortes et al., 2020). At present, it was the main method to predict DILI by measuring the physiological indexes of patients. Li et al. (2019) performed liver biopsy and common indicator tests in 465 patients, including blood lipid measurement, and the results suggested that dyslipidemia and female gender significantly increased the risk of DILI. Zhang et al. (2018) analyzed the toxic effects of 34 KIs on primary rat and human hepatocytes and further performed the prediction effect of KI-induced clinical hepatotoxicity, but the accuracy might be low (65% with human hepatocytes and 59% with rat cells).

It is well-known that the properties of drugs are the important factors for the increase of accuracy in DILI prediction. In earlier studies, Lammert et al. (2008) found a correlation between defined daily dose (DDD) ≥ 50 mg/day and severe DILI (death or liver transplantation). The octanol–water partition coefficient (LogP) affects the absorption of drugs and was also considered to be linked with DILI. Chen et al. (2013) explored the relationship between DDD and LogP and then put forward “rule of two” with a positive rate of 85%. However, their data set is relatively small, accounting for only around 30% of all drugs approved by the FDA. Weng et al. (2015) expanded the data set and concluded that the joint prediction between DDD and LogP was no better than using DDD alone, and it was proposed that the extensive liver metabolism contributes to the prediction of DILI. Subsequently, Chen et al. (2016) added the factor of active metabolite production to the “rule of two” rule, which improved the accuracy of prediction. Other drug properties that affect DILI included the changes in mitochondrial function (Han et al., 2013), inhibition of the bile salt export pump (Aleo et al., 2014), and liver transporter inhibition (Morgan et al., 2013). It can be seen that DDD, LogP, liver metabolism, and other drug properties are significantly related to DILI and can be used for the prediction of DILI. In addition, studies have shown that molecular weight (MW) and total polar surface area are related to drug adverse reactions (Hughes et al., 2008). However, further exploration on MW and the combination of multiple drug properties are still needed to understand and identify the DILI association at a relatively larger data set.

The mechanism of DILI is a complex process. Although the specific mechanism is still unclear, more and more studies show that mitochondria play a key role (Pessayre et al., 2012; Aleo et al., 2014). Researchers found that drugs or their active metabolites covalently bound to mitochondria, increased mitochondrial oxidative stress (ROS and RNS), damaged the mitochondrial DNA and protein, finally, resulted in mitochondrial dysfunction and the following cell necrosis and/or apoptosis (Jaeschke et al., 2012; Pessayre et al., 2012; Ye et al., 2018). Further studies showed drugs that induced mitochondrial dysfunctions could be used to predict DILI occurrence in humans (Porceddu et al., 2012; Aleo et al., 2014). However, few studies on mitochondrial toxicity of KIs were available. Recently, Zhang et al. (2017a) reported that the DILI mechanism might be related to the mitochondrial toxicity by measuring the effects of 34 FDA-approved KIs on the mitochondrial functions of rat primary hepatocytes. Perhaps due to the limitation of experimental conditions, it is difficult to carry out a large-scale study on the associations between all FDA-approved KIs and the mitochondrial toxicity using the traditional methods of molecular biology, especially how to find KIs closely bound to some important proteins in numerous mitochondrial proteins. *In silico* research methods should be a better alternative. In recent years, molecular docking has become a reliable tool for high-throughput screening of drug candidates and prediction of clinical adverse reactions, because of its low cost and simplicity. Chen and Ung (2001) used a ligand–protein reverse docking method to predict the adverse drug reactions (ADRs) and related target proteins, and 83% of the predicted results were consistent with the experimental results. Another similar study (LaBute et al., 2014) predicted some drug-related adverse reactions by calculating docking scores about drugs bound to protein targets, and the relative results were supported by PubMed literature. Furthermore, Jaundoo et al. (2018) used three different programs of docking to predict the hypothetical adverse reactions, and indicated that several FDA-approved drugs for the treatment of Gulf War Illness should be used with caution due to their high binding potential with immune and hormonal targets. Nevertheless, there are few studies using molecular docking to predict the potential of KI-induced liver injury. Through high-throughput docking of drugs with DILI related mitochondrial proteins may be able to analyze the potential and key proteins of DILI. Thus, the high-throughput docking between drugs and mitochondrial proteins may be helpful for both the understanding of KI-induced mitochondrial dysfunctions and related mechanism of DILI.

In this study, we collected information on FDA-approved oral drugs including small molecule KIs, and analyzed the DILI potential and drug characteristics through statistical methods and high-throughput molecular docking is achieved between nearly 95% of drugs in our data set and mitochondrial proteins. As a result, we found a multi-factor rule that may effectively predict DILI and the binding affinity between KIs and those mitochondrial proteins was significant. It may be helpful to further test this rule during drug development and clinical settings and explain the DILI mechanism of KIs.

## Materials and Methods

### Data Collection and Processing

We collected FDA-approved oral drugs through three databases, namely, PubChem (Kim et al., 2018), DrugBank (Wishart et al., 2018), and the World Health Organization (WHO). The drug structures and properties were collected from PubChem and DrugBank, and the Anatomical Therapeutic Chemical (ATC) codes and defined daily doses (DDD) (as of 1 September 2020) were harvested from the WHO website. If a drug has several DDDs reported corresponding to different body weights, we used the average value as the final value. The lipophilicity of drugs was calculated by the octanol–water partition coefficient (LogP) through ALOGPS 2.1 (Chen et al., 2013; Weng et al., 2015).

### KI Drug Information Collection

The WHO updates the approved drug information every year. As of the date when drug information was collected in this study (i.e., 1 September 2020), 53 KI drugs were retrieved in the WHO. One non-oral drug and 8 drugs for which liver side effect information could not be obtained were removed (the specific possible reasons for the lack of information were listed in Supplementary Table S1), and finally 44 KI drug information were obtained for analysis.

### Endpoint Collection and Grouping

Referring to the label classification method of Chen et al. (2011) and Liu et al. (2020), we collected seven DILI endpoints (fatality, liver failure, liver transplantation, hepatitis, hepatomegaly, jaundice, and abnormal biomarkers) and liver metabolism (LM) extent from Micromedex Drugdex (Solutions, 2018) and DailyMed (https://dailymed.nlm.nih.gov/dailymed/index.cfm) as the official website of FDA providing the reliable information about drug labels. Although there were vague descriptions in a few drugs related to liver injury, it was verified by the following database: Micromedex DrugPoints, LiverTox (Hoofnagle et al., 2013), and Hepatox (Mao, 2014). The drugs were divided into three data groups: severe DILI, less-severe DILI, and no DILI according to the endpoints (Chen et al., 2011; Liu et al., 2020). The severe DILI group included drugs that caused the fatality, liver failure, and liver transplantation in accordance with Hy’s law, issued by the FDA with a black box warning or withdrawn from the market. The less-severe DILI group included drugs that caused hepatitis, hepatomegaly, jaundice, and abnormal biomarkers, which are moderate DILI and can be generally improved by stopping the drug use. The no DILI group included drugs with no description of DILI on the labels. We ended up collecting 1,223 drugs (Supplementary Table S2), including 283 severe DILI drugs, 322 less-severe DILI drugs, and 618 no DILI drugs.

In order to further verify the reliability of collected DILI information in this study, one database reported by Teschke and Danan (2020) was used to compare with our data. A total of 81,856 DILI cases in the database were assessed using the Roussel Uclaf Causality Assessment Method (RUCAM) as the scale for quantitative evaluation of the causal relationship between drugs and liver injury, and these cases were involved in 220 drugs. By comparison, 154 (70%) of 220 drugs were found hepatotoxic (100%) in our data (details were shown in Supplementary Table S7), and the rest were excluded, mainly due to two reasons, one was not approved by FDA, and the other was a not oral drug.

### Molecular Docking

#### Human Mitochondrial Protein Collection and Processing

The crystal structures of human mitochondrial proteins were harvested using the RCSB PDB database (Berman et al., 2000). Proteins with different PDB entries may have different binding sites, so we keep the protein structure under each entry. All the proteins were visualized and processed using BIOVIA Discovery Studio 2019 to remove all the water molecules. For proteins with multiple subunits, if the active site was between the subunits, the whole protein was retained; otherwise, only one of the subunits was retained. For proteins with ligands embedded, the two parts were spared and stored separately. We utilized AutoDock Tools (ADT) 1.5.6 (Morris et al., 2009) to add hydrogen atoms to proteins, and then converted them into the pdbqt format.

#### Cytochrome P450 Protein Collection and Processing

The crystal structures of cytochrome P450 proteins were harvested using the RCSB PDB database (Berman et al., 2000). We collected 7 important CYP450 family enzymes structures (CYP3A4, CYP3A5, CYP2C9, CYP2E1, CYP2C19, CYP1A2, and CYP2D6 mainly involved in the metabolism of drugs) according to the previous reports (Yu et al., 2014; Teschke and Danan, 2021a). The pretreatment method was the same as the mitochondrial protein mentioned before.

#### Drug Molecule Processing

Drugs with 3D sdf files were downloaded from PubChem and converted into the pdbqt format.

#### Docking Parameter Setting

The originally embedded ligands of proteins were used as references to set the center of Grid Boxes through ADT. For proteins without ligands, AutoLigand and the structural reports of the proteins were used to identify the binding pocket (Morris et al., 2009). All the docking pockets were set to have spacing = 1 Å. The grid box was set to a size equivalent or larger than the largest molecule within the molecules we collected to ensure a sufficient size for docking.

#### Docking and Result Processing

AutoDock Vina was used for molecular docking (Trott and Olson, 2010), and the best docking score was selected for each ligand–protein pair. After removing the missing values, a total of 1,159 drugs (Supplementary Table S3) and 187 proteins (Supplementary Table S4) were left for analysis. In order to improve the comparability of the docking results, we normalized the docking result matrix using the following formula (Yang et al., 2009).

$$Z_{\mathrm{ij}}=\frac{X_{\mathrm{ij}}\;-\;\bar{X_{J}}}{SD_{X_{j}}},$$

$$\bar{X_{J}}=\frac{\sum_{i=1}^{N_{j}}X_{\mathrm{ij}}}{N_{j}},$$

$$SD_{X_{J}}=\sqrt{\frac{\sum_{i\mathrm{=1}}^{N_{j}}{\left(X_{\mathrm{ij}}-\bar{X_{J}}\right)}^{2}}{N_{j}\;-\;1}}.$$

### Molecular Dynamics Simulation

The whole molecular dynamics simulation was carried out using GROMACS packages (http://www.gromacs.org/). Proteins and drug small molecules were constructed using charmm36 force field version 2019 (http://mackerell.umaryland.edu/charmm_ff.shtml). The missing atoms of amino acid residues were completed using software SPDBV (https://spdbv.unil.ch/), and the coordinate files and topology files of proteins were generated using GROMACS. Adding hydrogen atoms to small molecules, and then CGENFF server was used (https://cgenff.umaryland.edu/) to obtain small molecule coordinate files and topology files. After the coordinate files and topological files of proteins and small molecules were combined, a dodecahedral unit cell 1 nm larger than the complex was defined and water molecules were added. The energy of the solvation system was minimized, and the topological files and coordinate files of the whole solvent system were generated. In order to ensure that the net charge was 0, Na + or Cl- need to be added to the box. Before the final simulation, the energy of the whole system needed to be minimized. The two energy minimization processes were limited to 50,000 steps, and when the maximum force was less than 10 kJ/mol, the minimization stopped. Then the temperature and pressure were balanced in 100 ps, and the system temperature was controlled at 300 K. Finally, the molecular dynamics simulation of 10 ns was carried out, and the temperature of the system was controlled at 300 K. The root mean square deviation (RMSD) for evaluating the binding stability of protein/drug complex was calculated per frame (10 ps) for analysis.

### Statistical Analysis

To identify the association between DILI and drug properties, we calculated the odds ratios (OR) and *p*-values using Fisher’s exact test. ANOVA was used for the analysis and comparison of docking results. The Kruskal–Wallis test was used to analyze the relationship between DILI and mitochondrial protein binding.

## Results

### DILI Analysis

#### Drug Categories and DILI

The Anatomical Therapeutic Chemical (ATC) codes are a system of codes developed by the World Health Organization (WHO) that were assigned to drugs according to their indications or mechanisms of action. We analyzed the associations between drug categories defined by the first to fourth levels of ATC codes and the seven DILI endpoints for all 1,223 FDA-approved oral drugs. Odds ratios (OR) and significance of each association through Fisher’s exact test were calculated. Supplementary Figure S1 showed the odds ratios between different levels of ATC codes and DILI for only the relationships that have statistical significance. From this figure, the highest odds ratios toward DILI were found in antineoplastic and immunomodulating agents (ATC code: L, OR = 9.45, 95% CI = 5.00–17.87, *p* = 6.35E-18), antineoplastic agents (ATC code: L01, OR = 17.41, 95% CI = 6.29–48.16, *p* = 1.80E-15), other antineoplastic agents (ATC code: L01X, OR = 17.94, 95% CI = 5.56–57.89, *p* = 1.67E-12), and kinase inhibitors (KIs) (ATC code: L01XE, OR = 46.89, 95% CI = 6.44–341.63, *p* = 9.28E-13) for the first to fourth levels of ATC codes, respectively. Detail information of Supplementary Figure S1 was shown in Supplementary Table S5. It is worth mentioning that KIs are shown to have high DILI potential in this analysis.

#### Drug Categories and Severe DILI

Severe DILI included outcomes of death, liver failure, and liver transplantation, which may lead to a black box warning or even withdrawal of the drug. We analyzed the relationship between different levels of ATC codes and severe DILI and showed the results in Supplementary Figure S2. It is observed that while the rankings and values of ATC codes with OR > 1 are slightly different from Supplementary Figure S1, the top ones in all the levels are consistent. The highest odds ratios toward severe DILI were found in antineoplastic and immunomodulating agents (ATC code: L, OR = 12.38, 95% CI = 6.35–24.14, *p* = 4.70E-18), antineoplastic agents (ATC code: L01, OR = 21.60, 95% CI = 7.59–61.39, *p* = 1.30E-14), other antineoplastic agents (ATC code: L01X, OR = 22.44, 95% CI = 6.76–74.46, *p* = 5.61E-12), and KIs (ATC code: L01XE, OR = 62.21, 95% CI = 8.40–460.94, *p* = 7.57E-13) for the first to fourth levels of ATC codes, respectively. The odds ratio of KIs increased from 46.89 for DILI to 62.21 for severe DILI, indicating their high potential to cause severe DILI in comparison with other types of drugs. Detail information of Supplementary Figure S2 was shown in Supplementary Table S6.

### Analysis of Kinase Inhibitors

#### KI Statistics on DILI

**FIGURE 1:**
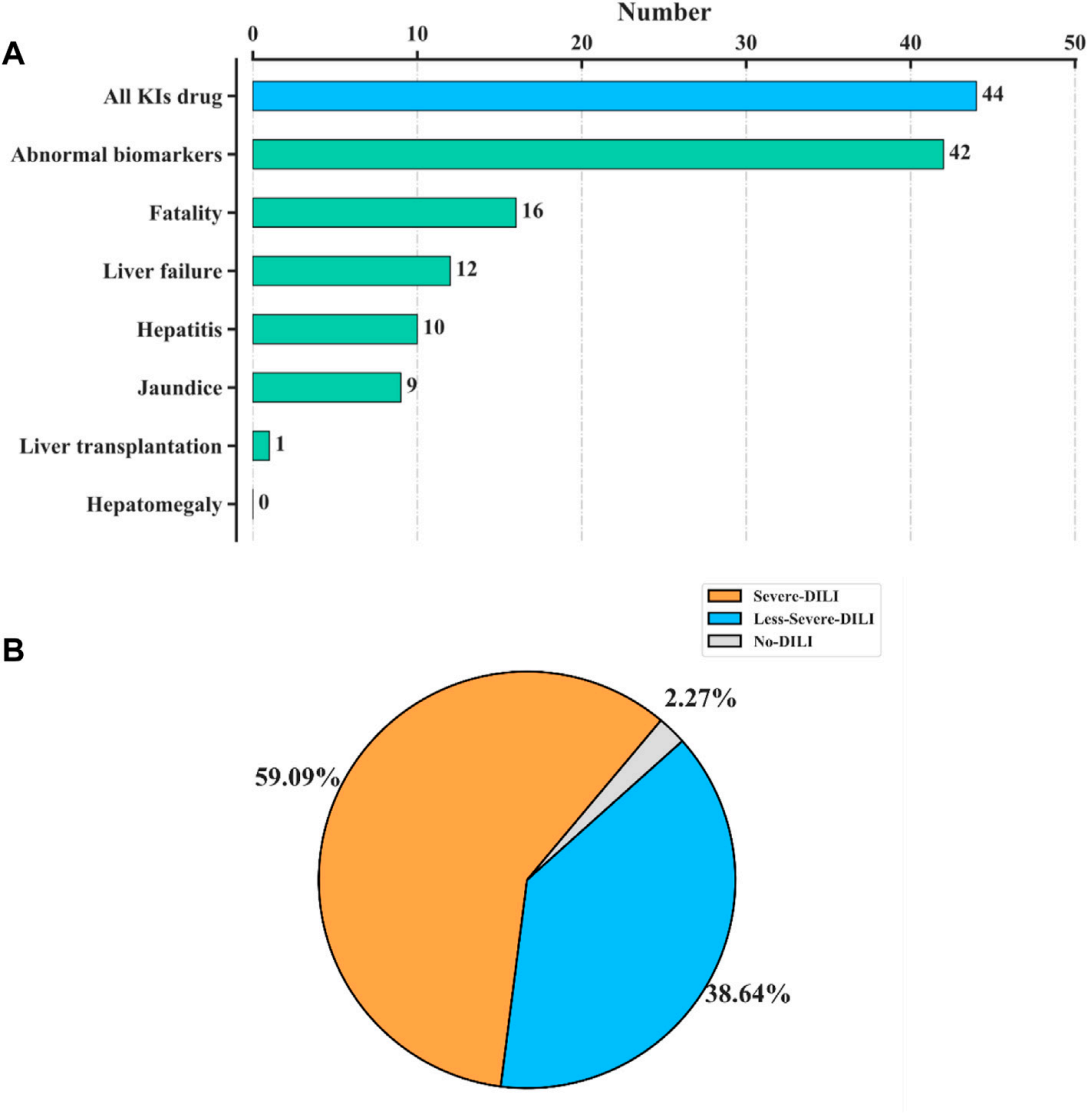
Statistics of 44 kinase inhibitors (KIs) on DILI. **(A)** Numbers of different DILI endpoints reported in KIs. For the 44 KIs collected in our data, 16 of them may cause death, and 12 may lead to liver failure. **(B)** Pie chart of the 44 KIs by three DILI severeness categories, “severe DILI,” “less-severe DILI,” and “no DILI.” A total of 43 of the 44 KIs (97.7%) were reported with DILI, while 26 (59.09%) may lead to severe DILI.

As of 1 September 2020, there were 53 KI drugs retrieved from the WHO. Excluding 1 non-oral drug and 8 drugs for which the information on liver side effects cannot be obtained (the specific possible reasons for the unavailability of information were shown in Supplementary Table S1), there were 44 available KI drug information. The statistics of different DILI endpoints caused by kinase inhibitors (KIs) were shown in Figure 1. For the 44 KIs, 16 of them may cause death and 12 may lead to liver failure (Figure 1A). In addition, 43 of the 44 KIs (97.7%) were reported with DILI, while 26 (59.09%) may lead to severe DILI (Figure 1B), which indicated a close link between KIs and serious DILI.

### Analysis of KI Properties

**FIGURE 2:**
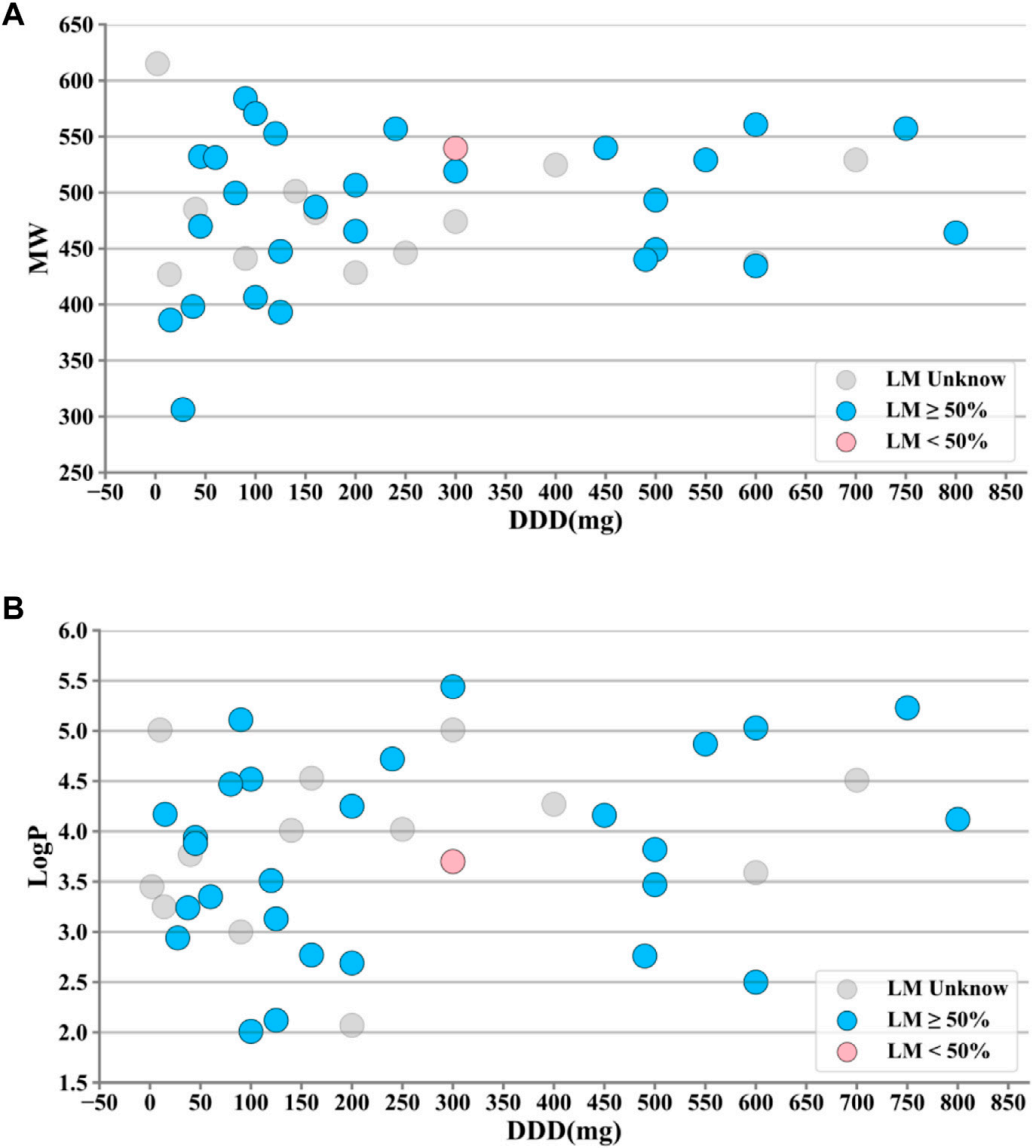
Property distributions of 44 kinase inhibitors (KIs). **(A)** Distributions of defined daily dose (DDD) and molecular weight (MW) values along with liver metabolism (LM) markups. **(B)** Distributions of DDD and octanol–water partition coefficient (LogP) values along with LM markups. It was observed that the majority of KIs met the criteria of LogP ≥ 3, DDD ≥ 100mg, and 400 ≤ MW < 600. Additionally, the majority of KIs (29 out of 44) have a known degree of liver metabolism (LM) greater than 50%, while only one KI has a known degree of liver metabolism (LM) less than 50%. #A few outliers with large deviations were removed.

The drug properties of KIs, including LogP, defined daily dose (DDD), liver metabolism (LM), and molecular weight (MW), were analyzed individually or in combination with DILI occurrence (Figure 2). It was observed that the majority of KIs met the criteria of LogP ≥ 3, DDD ≥ 100mg, and 400 ≤ MW < 600. Additionally, the majority of KIs (29 out of 44) have a known degree of liver metabolism (LM) greater than 50%, while only one KI has a known degree of liver metabolism (LM) less than 50%.

### Rules Associated With DILI

We combined KIs along with the remaining 1,179 drugs to explore the factors that may be highly associated with DILI. The different combinations of MW, LogP, DDD, and LM were tested and the results were shown in Table 1. Of all the rules, the association reached the highest when using the rule of “400 ≤ MW < 600 and LogP ≥ 3 and DDD ≥ 100 and LM ≥ 50%” (OR = 8.28, 95% CI = 2.46–27.82, *p* = 4.82E-05, predictive positive rate [PPV] = 88.00%).

**TABLE 1:** Associations between different risk criteria and drug-induced liver injury (DILI).

Criteria		DILI		OR (95% CI)	PPV% (%)
		Y	N		
LogP ≥ 3	Y	229	210	1.32^*^ (1.04–1.67)	52.16
	N	335	405		
DDD ≥ 100	Y	339	289	1.70^***^ (1.35–2.14)	53.98
	N	225	326		
LM ≥ 50%	Y	324	247	2.01^***^ (1.59–2.54)	56.74
	N	240	368		
LogP ≥ 3 and DDD ≥ 100	Y	130	72	2.26^***^ (1.65–3.09)	64.36
	N	434	543		
LogP ≥ 3 and LM ≥ 50%	Y	148	114	1.56^**^ (1.19–2.06)	56.49
	N	416	501		
DDD ≥ 100 and LM ≥ 50%	Y	186	92	2.80^***^ (2.11–3.71)	66.91
	N	378	523		
LogP ≥ 3 and DDD ≥ 100 and LM ≥ 50%	Y	84	34	2.99^***^ (1.97–4.53)	71.19
	N	480	581		
400 ≤ MW < 600 and LogP ≥ 3 and DDD ≥ 100 and LM ≥ 50%	Y	22	3	8.28^***^ (2.46–27.82)	88.00
	N	542	612		

Data were collected from Micromedex Drugdex, Micromedex DrugPoints, DrugBank, DailyMed, LiverTox, Hepatox, and PubChem databases. *p* value was calculated by Fisher’s exact test. Y, positive; N, negative; OR, odds ratio; CI, confidence interval; LogP, octanol–water partition coefficient; DDD, defined daily dose; LM, liver metabolism; MW, molecular weight; PPV, positive predictive value. PPV (%) = (true positives)/(total of positives). # only shows the rules that have significant associations with DILI. ***p < 0.001; **p < 0.01; *p < 0.05.

Similarly, we analyzed the associations between the rules and severe DILI (Table 2). The same rule “400 ≤ MW < 600 and LogP ≥ 3 and DDD ≥ 100 and LM ≥ 50%” still had the highest association (OR = 8.26, 95% CI = 2.25–30.26, *p* = 5.06E-04, predictive positive rate [PPV] = 76.92%). This rule is highly predictive against DILI and the severe subset of it.

**TABLE 2:** Associations between different risk criteria and severe DILI.

Criteria		Severe DILI		OR (95% CI)	PPV% (%)
		Y	N		
DDD ≥ 100	Y	166	289	2.06^***^ (1.52–2.78)	36.48
	N	91	326		
LM ≥ 50%	Y	155	247	2.26^***^ (1.68–3.05)	38.56
	N	102	368		
LogP ≥ 3 and DDD ≥ 100	Y	65	72	2.55^***^ (1.76–3.71)	47.45
	N	192	543		
LogP ≥ 3 and LM ≥ 50%	Y	71	114	1.68^**^ (1.19–2.36)	38.38
	N	186	501		
DDD ≥ 100 and LM ≥ 50%	Y	93	92	3.22^***^ (2.3–4.52)	50.27
	N	164	523		
LogP ≥ 3 and DDD ≥ 100 and LM ≥ 50%	Y	42	34	3.34^***^ (2.07–5.39)	55.26
	N	215	581		
400 ≤ MW < 600 and LogP ≥ 3 and DDD ≥ 100 and LM ≥ 50%	Y	10	3	8.26^***^ (2.25–30.26)	76.92
	N	247	612		

Data were collected from Micromedex Drugdex®, Micromedex DrugPoints, DrugBank, DailyMed, LiverTox, Hepatox, and PubChem databases. *p* value was calculated by Fisher’s exact test. Y, positive; N, negative; OR, odds ratio; CI, confidence interval; LogP, octanol–water partition coefficient; DDD, defined daily dose; LM, liver metabolism; MW, molecular weight; PPV, positive predictive value. PPV (%) = (true positives)/(total of positives). # only shows the rules that have significant associations with severe DILI. ***p < 0.001; **p < 0.01; *p < 0.05.

### Molecular Docking Analysis

#### Relationship Between DILI Occurrence and Mitochondrial Proteins Binding

**FIGURE 3:**
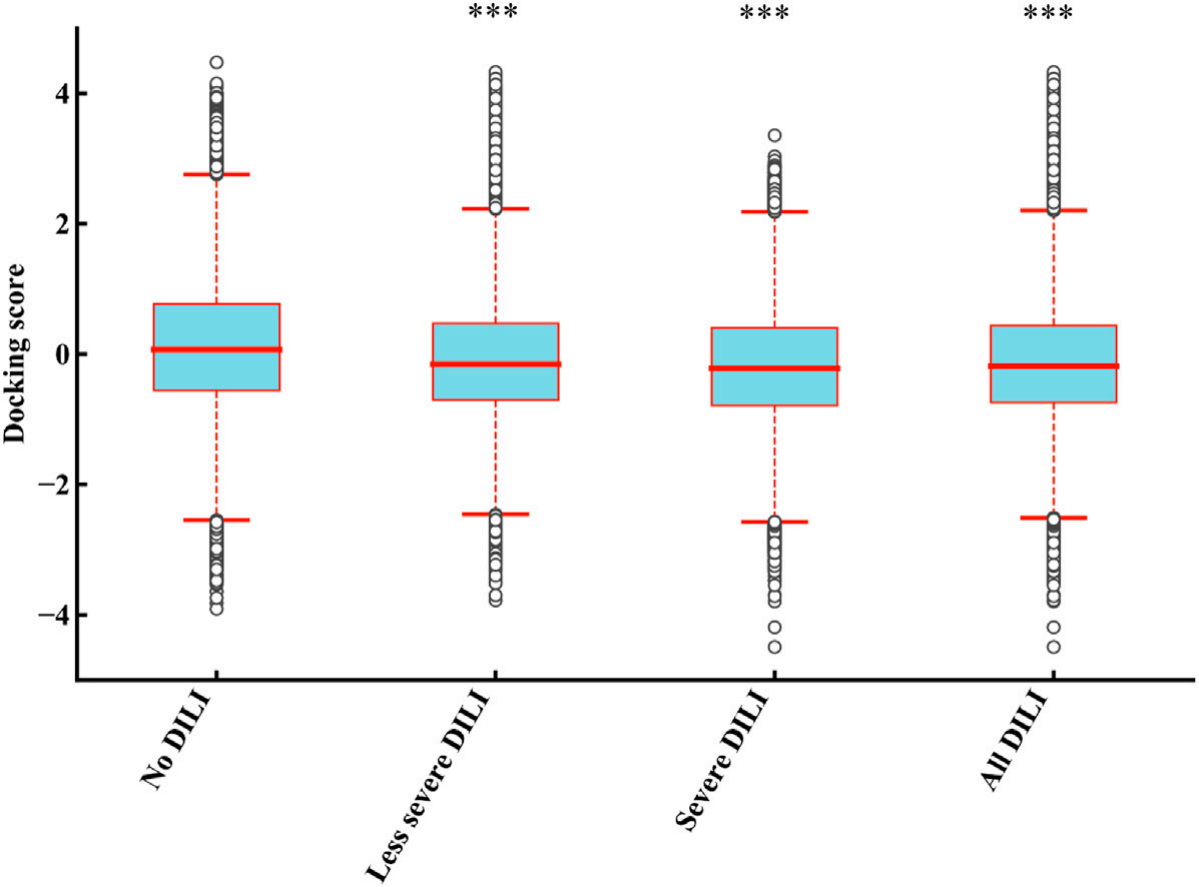
Comparison of results of 187 mitochondrial proteins binding between DILI groups and no DILI group. The docking scores of “less severe DILI,” “severe DILI,” and “all DILI” were significantly lower than those of the “no DILI” group (Kruskal–Wallis test). ***p < 0.001.

In order to determine the direct relationship between DILI and mitochondria proteins binding, we divided the 1,159 drugs into the four groups including “no DILI,” “severe DILI,” “less severe DILI,” and “all DILI,” and analyzed the binding affinity of these groups with 187 mitochondrial proteins. As shown in Figure 3, the docking scores of “less severe DILI,” “severe DILI,” and “all DILI” were significantly lower than those of the “no DILI” group, indicating that drugs with DILI had the better affinity to bind mitochondrial proteins (Kruskal–Wallis test). Also, it is worth noting that the affinity of the “severe DILI” group to bind mitochondrial proteins was also higher than that of the “less severe DILI” group.

#### Comparison of the Docking Results Across Drug Categories

1) Docking with human mitochondrial proteins.

**FIGURE 4:**
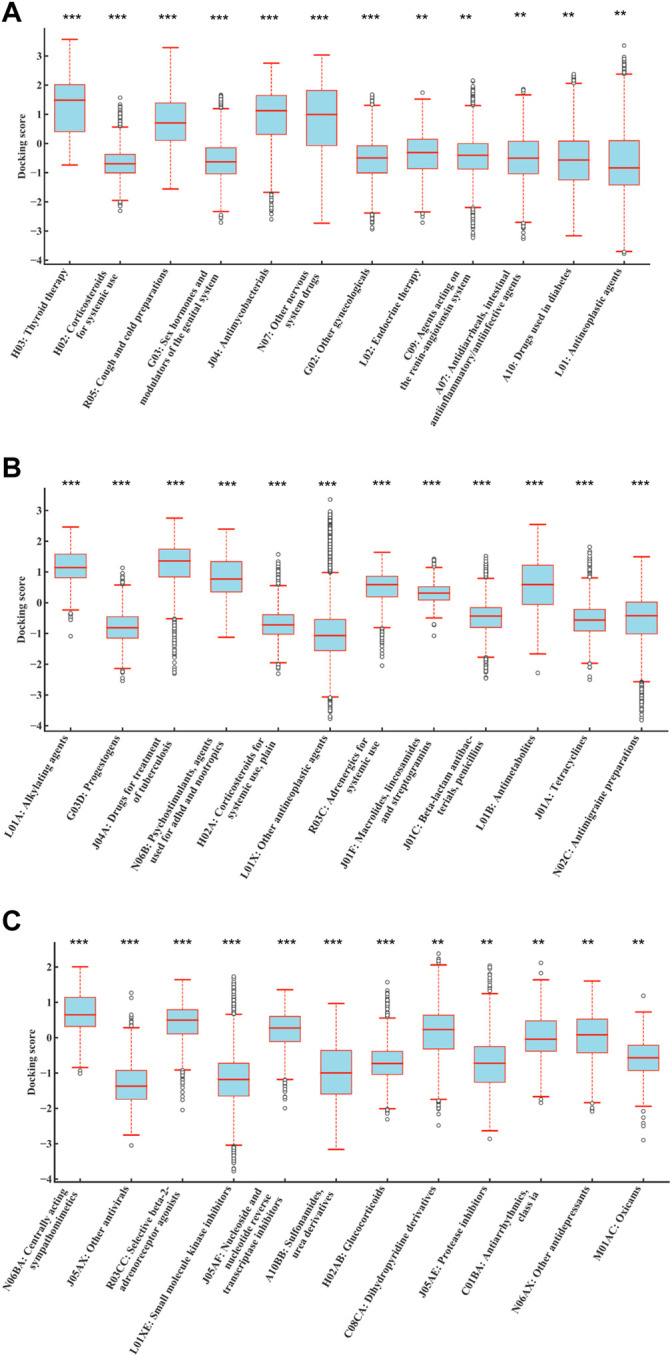
Distributions of normalized average docking scores of drugs categorized by different levels of ATC codes. The *y*-axis indicates the normalized average docking scores. The *x*-axis represents different ATC code categories. **(A–C)** represent ATC code levels from second to fourth. Drugs with ATC codes L01, L01X, and L01XE were all significantly different against other groups (*p* = 6.74E-03, 2.25E-08, and 4.19E-11, respectively) (ANOVA). It indicated that the result scores of KIs (ATC code: L01XE) were significantly better (lower in scores) than those of other types, which indicates that KIs may bind to mitochondrial proteins and cause DILI. #The results were sorted by significance from high to low, and only the first 12 were shown. ***p < 0.001; **p < 0.01; *p < 0.05.

1,159 drug molecules with available SDF structures were used to dock with 187 human mitochondrial proteins through AutoDock Vina, generating a 1,159 × 187 matrix with the result value normalized to a range between −4 and 4. For each drug, the average value of the docking scores with all the proteins was used as the result score. The result scores were grouped by their ATC code categories and compared against the rest using ANOVA. As shown in Figure 4, drugs with ATC codes L01, L01X, and L01XE were significantly different from other groups (*p* = 6.74E-03, 2.25E-08, and 4.19E-11, respectively). It was observed that the result scores of KIs (ATC code: L01XE) were significantly better (lower in scores) than other types, which indicates that KIs may bind to mitochondrial proteins and cause DILI.

2) Docking with Cytochrome P450 proteins.

**FIGURE 5:**
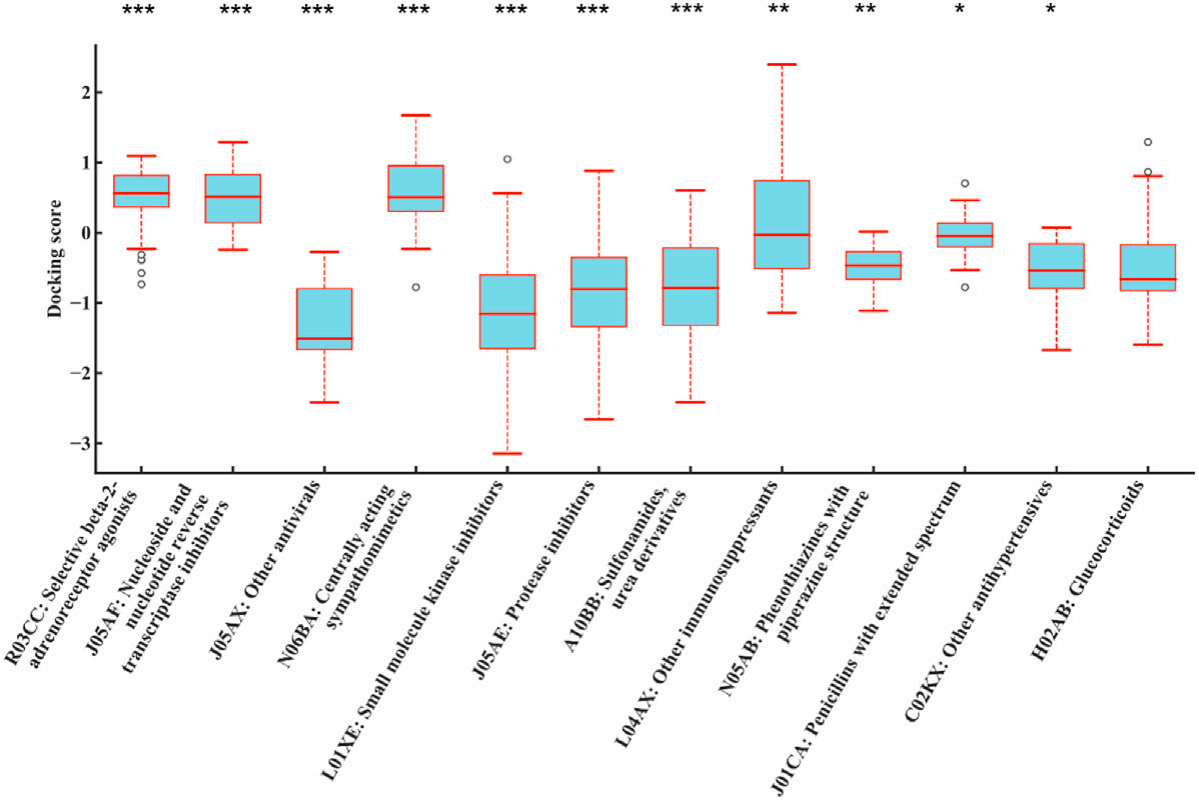
Distributions of normalized average docking scores between different drug class and 7 CYP family enzymes (CYP3A4, CYP3A5, CYP2C9, CYP2E1, CYP2C19, CYP1A2, and CYP2D6). The *y*-axis indicates the normalized average docking scores. The *x*-axis represents different ATC code categories. Drugs with ATC code L01XE were all significantly different against other groups (*p* = 1.07E-09) (ANOVA). It indicated that the result scores of KIs (ATC code: L01XE) were significantly better (lower in scores) than those of other types, which indicates that KIs may bind to CYP family enzymes and metabolized thus increased potential for DILI. #The results were sorted by significance from high to low, and only the first 12 were shown. ***p < 0.001; **p < 0.01; *p < 0.05.

A total of 1,159 drug molecules were used to dock with 7 CYP proteins through AutoDock Vina, generating a 1,159 × 7 matrix with the result value normalized to a range between -4 and 4. For each drug, the average value of the docking scores with all proteins was used as the result score. The result scores were grouped by their ATC code categories and compared against the rest using ANOVA. As shown in Figure 5, drugs with ATC code L01XE were significantly different from other groups (*p* = 1.07E-09). It was observed that the result scores of KIs (ATC code: L01XE) were significantly lower than other types, which indicates that KIs may have better affinity to bind to CYP enzymes.

### Score Analysis Between Mitochondrial Proteins and KIs

Since KIs have a higher chance to induce DILI compared to other drugs, it is important to identify which mitochondrial proteins may be associated with this. The top five proteins with the lowest docking scores of KIs and functions of these proteins were shown in Table 3. It can be seen that the protein with the lowest score is 3B96 (PDB ID), followed by 5FS8, 4FDH, 5G5J, and 6G2M, which may be of great help to the in-depth study of the mechanism of KI drugs DILI and the prevention of DILI. It should be noted the results were obtained by the average docking results of each protein with 44 KIs, and then the top five proteins with the lowest scores were selected after sorting. The comparison calculation process was carried out after normalization.

**TABLE 3:** Functions of the protein with the lowest binding score for kinase inhibitor (KI) drugs.

Protein PDB ID	Docking score mean ± SD	Name	Function
3B96	−1.65 ± 0.53	Very-long-chain acyl-CoA dehydrogenase (VLCAD)	A homodimer related to mitochondrial membrane (McAndrew et al., 2008)
5FS8	−1.59 ± 0.55	Oxidoreductase encoded by the AIFM1 gene	The apoptosis-inducing factor in the mitochondrion, which plays a key role in the energy metabolism process and is important for cell death (Sevrioukova, 2016)
4FDH	−1.53 ± 0.54	Aldosterone synthase	The only enzyme in humans producing aldosterone and plays an important role in regulating the balance of electrolyte and the pressure of blood (Strushkevich et al., 2013)
5G5J	−1.52 ± 0.39	Human CYP3A4	Related with eicosapentaenoic acids (EETs) that promote electron transport chain/respiration (Guo et al., 2017)
6G2M	−1.50 ± 0.48	Human mitochondrial 5′ (3′)-deoxyribonucleotidase	It can regulate the nucleotides and nucleosides pool in cell (Pachl et al., 2018)

### Relationship Between Five Key Proteins and DILI Occurrence

**FIGURE 6:**
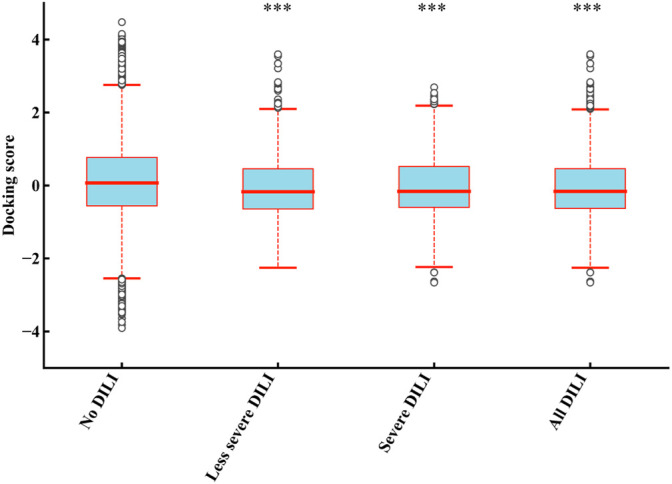
Comparison of results of five mitochondrial proteins (PDB ID: 3B96, 5FS8, 4FDH, 5G5J, and 6G2M) binding between DILI groups and no DILI group (KIs were not included). The docking scores of “less severe DILI,” “severe DILI,” and “all DILI” were significantly lower than those of “no DILI” group (Kruskal–Wallis test). ***p < 0.001.

To illustrate whether drugs with DILI can bind more strongly to the five proteins than drugs without DILI, we further analyzed the docking results of drugs excluding KIs with these five proteins (Figure 6). Compared with the “no DILI” group, the binding scores of the “All DILI” group were significantly lower, and indicated the affinity was better. Moreover, we further found that the affinity of the “severe DILI” group was higher than that of the “less severe DILI” group (Kruskal–Wallis test).

### Docked Conformations of Five Key Proteins and KIs

In order to further explore the binding sites on these five key proteins, we analyzed the docked conformations of five key proteins and KIs. The overall binding region was shown in Supplementary Figure S3. According to the formation of interaction bonds (mainly hydrogen bonds), some amino acids (marked in red) were considered as key interaction sites, such as protein 3B96: Thr177, Ser211, and Gly423; 4FDH: Pro442, Val378, and Gly379 ; 5FS8: Ala397, Gly399, and Phe284; 5G5J: Thr224, Phe108, and Pro107; 6G2M: Phe75, Arg177, and Thr181. Some drugs are likely to bind to the five key proteins by these sites, which could result in mitochondrial toxicity.

#### Molecular Dynamics Simulation of Five Key Proteins and KIs

**FIGURE 7:**
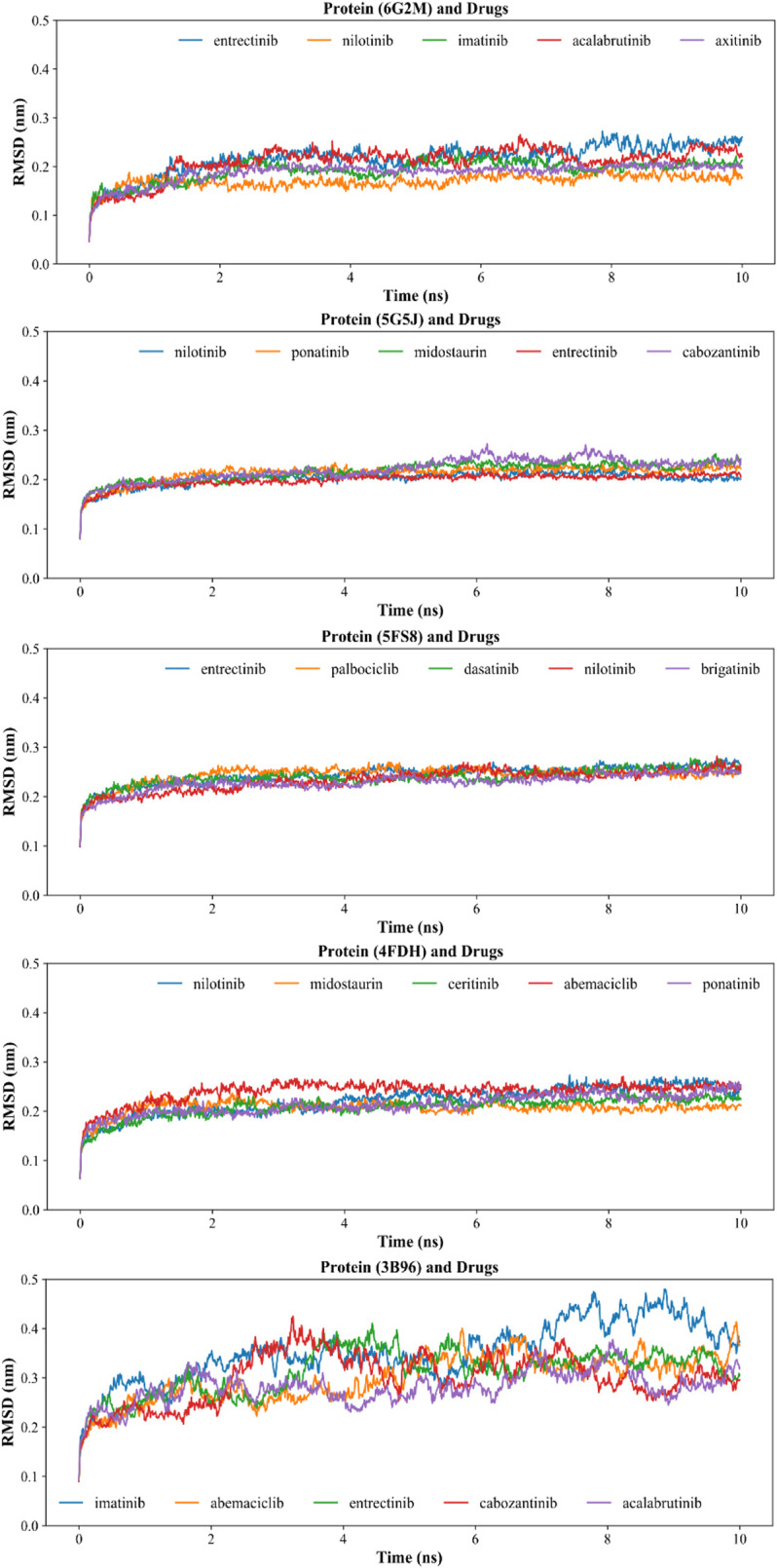
Backbone RMSD of molecular dynamics simulation of proteins and drug molecules. The codes in the title brackets were the PDB ID corresponding to the proteins in the complexes.

In order to further optimize and verify the reliability of the molecular docking results, we sorted the docking energy of all KIs and five key proteins, and selected the top five KIs with the lowest scoring for molecular dynamics simulation. The dynamic binding behavior of the protein/drug complex during 10 ns of simulation was studied by calculating the backbone root mean square deviation (RMSD), and total 25 pairs of complexes were simulated. As shown in Figure 7, among the five mitochondrial proteins, the smallest fluctuation of the backbone RMSD curve was found in the protein 5FS8/KIs complexes, which indicated the best stability of the protein 5FS8 bound to KIs. The average RMSD values of the complexes when protein 5FS8 interacted with brigatinib, nilotinib, dasatinib, palbociclib, and entrectinib were 0.229, 0.233, 0.239, 0.244, and 0.245 nm, respectively, and the results reflected that the combination of brigatinib was the most stable. It provided the reference significance for the further study of DILI mechanism of KIs.

According to previous studies, RMSD values < 0.3 nm indicated that the complex was a successful fit in the simulation process (Jorgensen et al., 2015; Bavi et al., 2020; Guterres and Im, 2020; Mishra and Rathore, 2021; Vishvakarma et al., 2022). Our results have shown that the RMSD values of other four proteins (4FDH, 5FS8, 5G5J, and 6G2M) interacting with KIs were chiefly between 0.15 and 0.25 nm, suggesting the majority of complexes were relatively stable except for the protein 3b96 interacting with imatinib which had a relatively larger mean RMSD of 0.35 nm. This may be related to the molecular size and structure of the drug (Wang et al., 2021). So, our results showed that the dynamic binding behavior of the selected most complexes (24/25) was stable, and also indicated that the conformations of our docking results in the previous step were reliable.

## Discussion

DILI is the main cause of acute liver failure (Sunil Kumar et al., 2021), and it is very important to predict and prevent DILI. We collected the information of drug properties and DILI endpoints of FDA-approved drugs and studied the risk factors toward DILI and the associations between KIs and DILI.

It has been reported that KIs have high DILI potential, and more than half of them cause DILI in clinic (Jiang et al., 2021). In our study, we found that KIs had the highest risk of DILI (OR = 46.89) among all drug categories. In addition, it should be noted that drugs with an OR < 1 do not mean that they had a protective effect on the liver, but they had a very small probability of causing liver toxicity than the control group. For these types of drugs, it may be possible in the future to find ways to reduce DILI by studying their mechanism, but this is only a preliminary idea, and further research and verification are needed.

For the reason of high DILI risk of KIs, we analyzed the common characteristics of KIs and found a rule that can contribute to a more efficient prediction of DILI in the validation by non-KI drugs. We first considered the reported DDD, LogP, and liver metabolism (Chen et al., 2013; Weng et al., 2015), and provided support for these drug properties can help to predict DILI. In addition, we also considered that WM played an important role in DILI prediction. On the other side, relative to the previous study (Lammert et al., 2008; Chen et al., 2013; Weng et al., 2015; Chen et al., 2016), this study was based on a larger data set. As a result (Table 1), when the drugs met “400 ≤ MW < 600 and LogP ≥ 3 and DDD ≥ 100 and LM ≥ 50%”, the risk of DILI was much higher than using any one of “logP ≥ 3’, “DDD ≥ 100” and “LM ≥ 50%” alone, or using them in combination with each other, especially 3.66 times as much as using “LogP ≥ 3 and DDD ≥ 100” (namely “rule of two”), and the OR value was also 3.78 times higher than that reported by Weng et al. (2015) based on the study of 975 oral drugs approved by the FDA. Also, the predictive positive rate increased from 85% reported by Chen et al. (2013) based on the study of 164 oral drugs approved by FDA to 88%. We also provided the effective prediction of severe DILI, and the OR value increased from 2.05 reported by Weng et al. (2015) to 8.26. In this rule, the factor MW played a vital role, and this was further evidence of previous reports in which MW was one of the determinants of the positive rate of DILI prediction (Yucha et al., 2017). This may help to predict DILI in advance and reduce the cost of early drug development.

On the other hand, the important role of MW in DILI prediction may be related to mitochondrial toxicity. In the study of Yu et al. (Zhang et al., 2017b), MW was one of the four key molecular descriptors used in the prediction model of drug-induced mitochondrial toxicity. Also, the direct relationship between mitochondrial toxicity and DILI occurrence has been gradually recognized (Meyers et al., 1988). Moreover, the recent study of Hemmerich et al. (2020) stated that chemicals with molecular weight between 250–600 may have significant effects on *in vitro* mitochondrial functions based on a large data set analysis of 5,761 compounds. In this study, we further found that the drugs of MW between 400 and 600, a smaller range, were closely related to the occurrence of DILI. Also, it was worth noting that our study may be more reliable in predicting human DILI, as it was based on the data of clinical trials and FDA adverse event reporting system.

It also suggests that the study of the binding between different kinds of drugs and mitochondria may explain the DILI potential and explore the key proteins bound with drugs. In our docking results, KIs had significantly higher binding affinities with mitochondrial proteins, and it suggested that KIs can be high potential of DILI, mainly due to KIs resulting in the mitochondrial toxicity (Zhang et al., 2017a; Jiang et al., 2021). On the other side, the statistic results also showed that drugs with DILI (especial severe DILI) were bound to mitochondrial proteins with higher affinity, on the whole, which suggested that a major reason for DILI occurrence was attributed to drug-induced mitochondrial dysfunctions.

Next, we found five key proteins with the highest binding affinity to KIs, namely, 3B96 (very-long-chain acyl-CoA dehydrogenase), 4FDH (aldosterone synthase), 6G2M (human mitochondrial 5′ (3′)-deoxyribonucleotidase), 5FS8 (oxidoreductase), and 5G5J (human CYP3A4). Also, the results of molecular dynamics simulation further verified the reliability of the docked conformations and interaction sites between KIs and the five key proteins, especially protein 5FS8 was the most stable when bound to KIs. It might provide a good chance to study the DILI mechanism related to KIs in future.

On the other hand, these proteins may play an important role in mitochondrial damage caused by compounds. Such as 3B96 is a homodimer related to the mitochondrial membrane and 4FDH can regulate electrolyte balance, and the out-of-balance of mitochondrial membrane potential is one of the mechanisms of DILI (Jaeschke et al., 2012; Oyebode et al., 2019); 5G5J is related to the regulation of electron transport chain, and the inhibition of 5G5J activity induces mitochondrial toxicity and thus leads to DILI (Chance and Hollunger, 1963; Hemmerich et al., 2020); 6G2M can regulate the levels of dTMP and dump to prevent excessive mutagenic dUTP from hindering the synthesis of mitochondrial DNA, and 5FS8 is an apoptosis-inducing factor. According to the previous study (Jaeschke et al., 2012), apoptosis-inducing factors cause DNA damage in the nucleus and then trigger cell death. These key proteins may be helpful for further understanding the mechanism of drug-induced mitochondrial toxicity and cause liver injury, but further basic experimentation is needed to address the issue.

It is worth noting that the DILI occurrence was related to high-intensity liver metabolism, and studies have shown that drugs related to liver injury were mainly metabolized by CYP450 (Tarantino et al., 2009; Teschke and Danan, 2021a). Our results of high-throughput docking between 1,159 drugs and 7 CYP450 enzymes also showed KIs (ATC code: L01XE) significantly tend to bind to main CYP enzymes against other drug types, suggesting that KIs had a higher affinity to CYP enzymes and had a higher potential to increase the DILI risk.

In this study, we expanded the data set and proposed a new rule for effectively predicting DILI. The most important was the factor of MW, which has been neglected, was incorporated into the rules of DILI prediction, and it was found that the combination of multiple conditions can effectively predict DILI.

But, our work also has certain limitations. First, the classification of relevant labels in the collection of drug data was artificial, and accidental errors are inevitable. Also, they are limited to oral medicines, and no reference can be provided for other forms of medicines such as injections. Second, RUCAM is a reliable evaluation method for DILI cases (Teschke and Danan, 2021b). In our study, 154 of 605 positive data were verified by RUCAM, based on the literature reported by Teschke and Danan (2020). However, it is not clear yet that there is any true causal relationship between liver injury and few drugs among the remaining drugs that have not been verified by RUCAM, especially less severe DILI drugs. Additionally, it is undeniable that the label information of few drugs is insufficient, such as the label information of some drugs shows no case report of liver toxicity due to short using period and smaller number of patients. Third, the mechanism of DILI occurrence is a very complex process involving many factors. Our study suggests that DILI is at least partly explained by a drug resulting in mitochondrial dysfunctions, but more research related to the mechanism is needed to address this issue. Finally, DILI has not been found in the relevant literature or reports of some drugs, especially newly approved drugs, but it may still cause DILI over time. In the future work, we will collect as much data as possible, increasing the sample size and feature amount, building a machine learning model to predict DILI, and conduct experimental verification of related conclusions through animal experiments.

## Conclusion

The drug properties and DILI endpoints of 1,223 FDA-approved oral drugs were collected, and different risk factors of DILI were analyzed. The risk of DILI is 8.28-fold higher for drugs that met rule “400 ≤ MW < 600 and LogP ≥ 3 and DDD ≥ 100 and LM ≥ 50%”. KIs were found to have the highest odds ratio of causing DILI among all ATC codes. The molecular docking results of 1,159 drugs and 187 mitochondrial proteins were studied, and the average docking scores of KI drugs were found to be significantly different from other classes. Further analysis identified the top binding mitochondrial proteins for KIs, and the sites interacting with KIs were obtained by docking conformations, and the stability of the complexes was verified by molecular dynamics simulation, which may contribute to studying the mechanism of DILI.

## Data Availability

The original contributions presented in the study are included in the article/Supplementary Material, further inquiries can be directed to the corresponding authors.
