# Supplementary material for: Study on the Characteristics of Small-Molecule Kinase Inhibitors-Related Drug-Induced Liver Injury
Source: Front Pharmacol. 2022 Apr 21;13:838397. doi: 10.3389/fphar.2022.838397 (PMC9068902; doi:10.3389/fphar.2022.838397)
Supplement: Supplementary file 3 [file Image1.pdf]

Supplementary materials

Study on the Characteristics of Small-molecule Kinase Inhibitors Related Drug-induced Liver Injury

Huiqun Dong [1, †], Jia You [3, †], Yu Zhao[2], Danhua Zhen [1], Yi Zhong [1, 2], Gaozheng Li [2], Zuquan Weng [1, 2, *], Heng Luo [1, 2, *], Shan Jiang [4, *]

[1] College of Biological Science and Engineering, Fuzhou University, Fujian province, China

[2] College of Mathematics and Computer Science, Fuzhou University, Fujian province, China

[3] Department of Hepatology, Hepatology Research Institute, the First Affiliated Hospital, Fujian Medical University, Fuzhou, Fujian province, China

[4] Department of Vascular Thyroid Surgery, Affiliated Union Hospital, Fujian Medical University, Fuzhou, Fujian province, China

[†] These authors contributed equally to this work.

*** Correspondence:**
Zuquan Weng
wengzq@fzu.edu.cn
Heng Luo
hengluo88@gmail.com
Shan Jiang
jiangshan001@fjmu.edu.cn

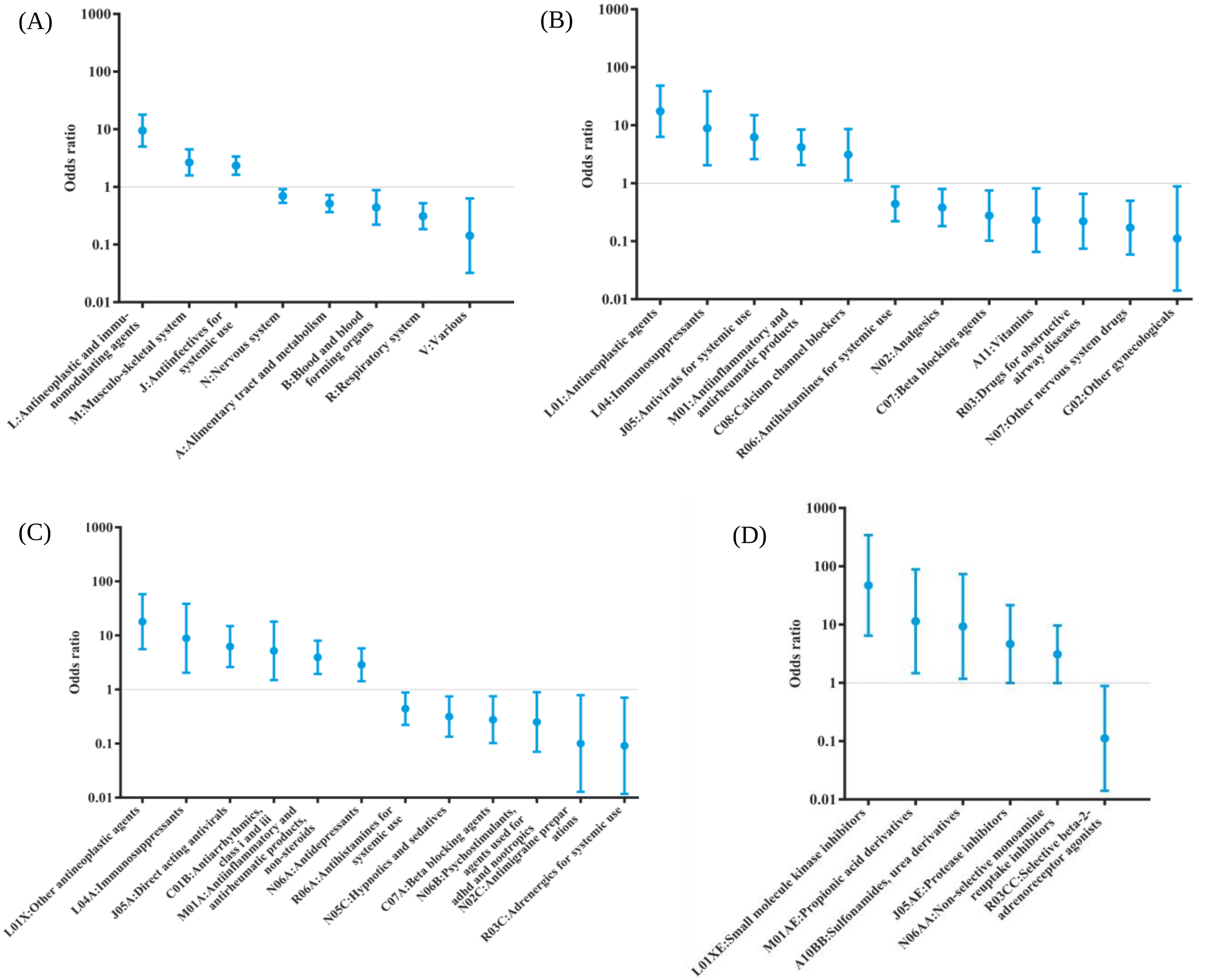

Figure S1. The odds ratios between different levels of the Anatomical Therapeutic Chemical (ATC) codes and DILI. (A)-(D) represent first to fourth levels of ATC codes. Only ATC codes with significant p values were shown in this figure (Fisher's exact test). The highest odds ratios towards DILI were found in L, L01, L01X, and L01XE for first to fourth levels of ATC codes, respectively. It is worth mentioning that KIs (ATC code: L01XE, OR = 46.89, P = 9.28E-13) are shown to have high DILI potential in this analysis.

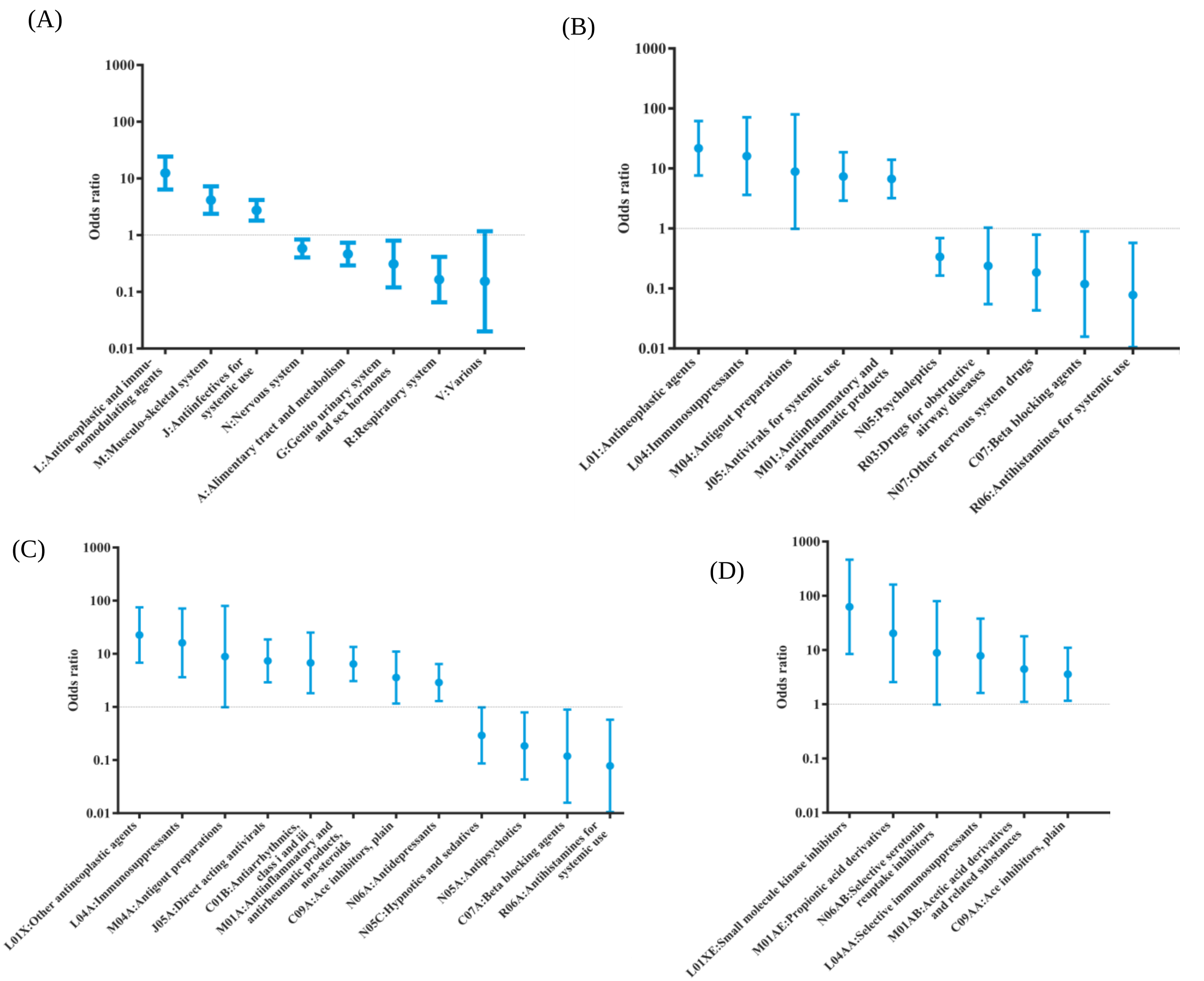

Figure S2. The odds ratios between different levels of ATC codes and Severe DILI. (A)-(D) represent first to fourth levels of ATC codes. Only ATC codes with significant p values were shown in this figure (Fisher's exact test). The highest odds ratios towards Severe DILI were consistent with the results of DILI. But the odds ratio of KIs (ATC code: L01XE, OR = 62.21, 95% CI = 8.40-460.94, *P* = 7.57E-13) increased from 46.89 for DILI to 62.21 for Severe DILI, indicating their high potential to cause severe DILI in comparison with other types of drugs.

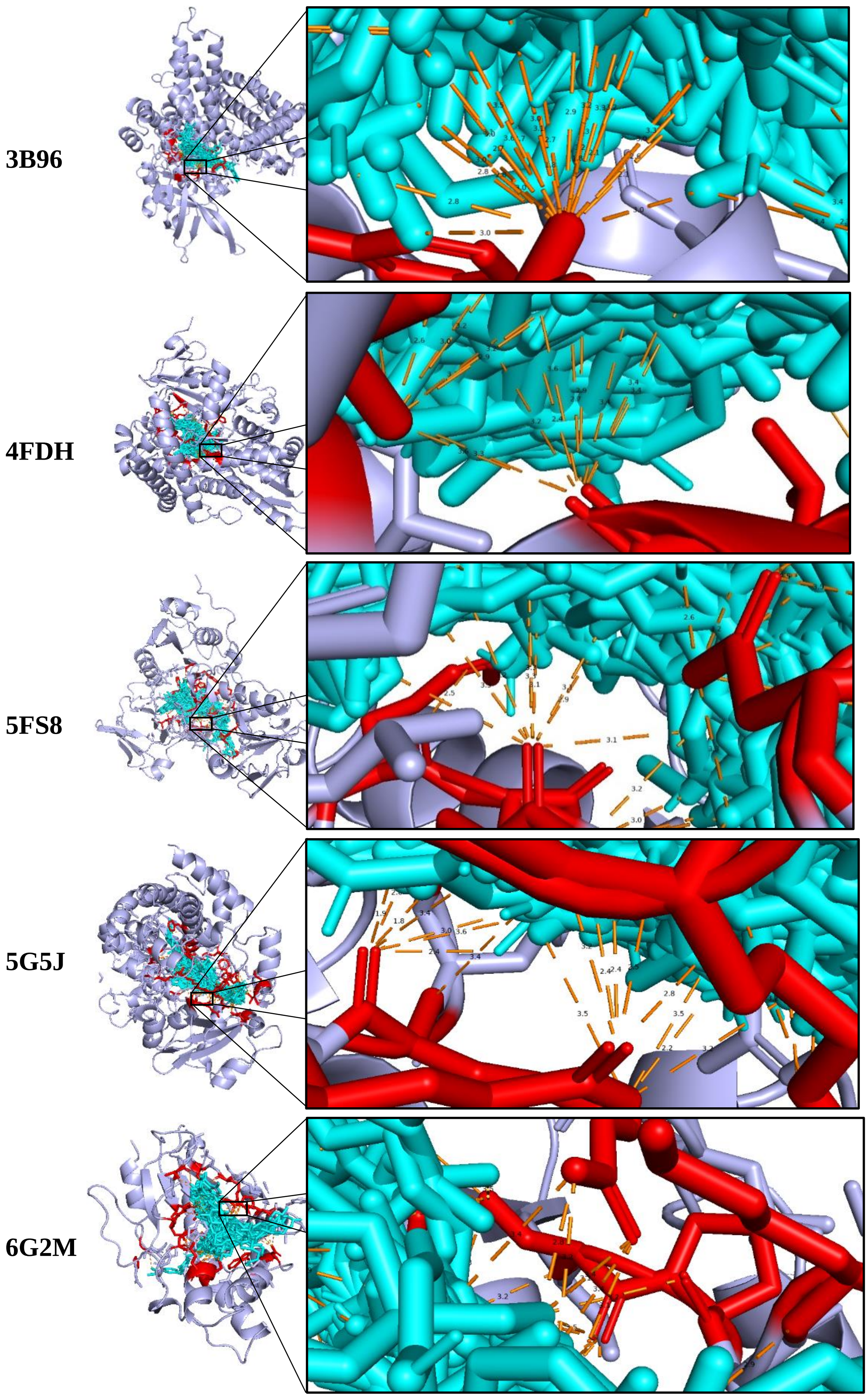

Figure S3. Docking conformations of five key proteins and KIs. Lilac marked protein, cyan marked drug molecule, orange marked hydrogen bond, and red marked amino acid residue with interaction.
